# Preliminary evaluation of the efficacy and safety of brimonidine for general anesthesia

**DOI:** 10.1186/s12871-021-01516-1

**Published:** 2021-12-03

**Authors:** Chen Bin, Wang Xiaohui, Shi Mengrou, Li Xin, Zhang Ting, Gao Ping

**Affiliations:** Tianjin Institute of Medical & Pharmaceutical Sciences, No. 79 Duolun Road, Heping District, Tianjin, 300020 China

**Keywords:** Brimonidine tartrate, Adrenergic alpha-2 receptor agonists, General anesthesia, Lethal dose 50, Effective dose 50

## Abstract

**Background:**

To determine the hypnotic and analgesic effects of brimonidine, and evaluate its efficacy and safety for general anesthesia. Potentiation of pentobarbital sleeping time following brimonidine administration was observed in mice, as was the analgesic activity of brimonidine.

**Methods:**

The median effective dose (ED_50_) and lethal dose (LD_50_) of intraperitoneally injected brimonidine were determined in hypnotized mice. In addition, the LD_50_ of intravenously injected brimonidine, and ED_50_ of intravenously, intramuscularly, and intrarectally injected brimonidine in hypnotized rabbits were determined. Finally, the synergistic anesthetic effect of brimonidine and chloral hydrate was evaluated in rabbits.

**Results:**

Intraperitoneal injection of 10 mg/kg brimonidine enhanced the hypnotic effect of a threshold dose of pentobarbital. Intraperitoneally injected brimonidine produced dose-related analgesic effects in mice. The ED_50_ of intraperitoneally administered brimonidine in hypnotized mice was 75.7 mg/kg and the LD_50_ was 379 mg/kg. ED_50_ values of intravenous, intramuscular, and intrarectal brimonidine for hypnosis in rabbits were 5.2 mg/kg, 8.8 mg/kg, and 8.7 mg/kg, respectively; the LD_50_ of intravenous brimonidine was 146 mg/kg. Combined intravenous administration of 0.6 mg/kg brimonidine and 0.03 g/kg chloral hydrate had a synergistic anesthetic effect.

**Conclusions:**

Brimonidine elicited hypnotic and analgesic effects after systemic administration and exhibited safety. Moreover, brimonidine enhanced the effects of other types of narcotics when combined.

**Supplementary Information:**

The online version contains supplementary material available at 10.1186/s12871-021-01516-1.

## Background

General anesthesia involves drugs that widely inhibit the central nervous system to temporarily inhibit consciousness and nerve response activities, such that the body has no response or memory to pain and noxious stimulation. Such effects may be accompanied by a decrease of muscle tension caused by central mechanisms, but the important central functional state of the medulla oblongata is maintained.

α_2_ Adrenergic receptor agonists, such as dexmedetomidine, are often used as adjuncts to clinical general anesthesia, but can also be used as a basic anesthetic in combination with local anesthetics for surgery. Such anesthetics act as both sedatives and analgesics, and may have hypnotic, anti-anxiety, anti-sympathetic, and muscle relaxation effects. Moreover, these anesthetics protect organs, have good safety profiles, are not addictive.α_2_ adrenergic receptor agonists are often used in combination with other anesthetics to meet the anesthesia needs of various surgical settings during the induction period, maintenance period, and awakening period. Dexmedetomidine and clonidine are already widely used for clinical anesthesia. Their characteristics include good hypnotic and analgesic effects, as well as the abilities to strengthen the efficacy of other central sedative and analgesic drugs, and reduce the dosage of other drugs (e.g., propofol, fentanyl, and isoflurane), thus reducing toxicity and side effects. Similar anesthetic effects could be expected using brimonidine.

In this study, we investigated the general anesthesia effects of brimonidine, a highly selective α_2_ receptor agonist with high activity and good water-fat solubility, and preliminarily evaluated its safety. At present, no reports describe induction or maintenance of general anesthesia by brimonidine alone or in combination. This study evaluated the analgesic and hypnotic potentiation of brimonidine systemic administration against various animal experimental models. Here, we report the first investigation into the general anesthesia activities of brimonidine.

## Methods

### Laboratory animals

Japanese white rabbits of ordinary grade (Longan Experimental Animal Breeding Center, Beijing, China), weighing 2.3–2.6 kg were used in experiments [Animal Production License No. SCXK (Beijing) 2019–0006]. In addition, specific-pathogen-free Kunming (KM) mice (Beijing Weitonglihua Experimental Animal Center, China), weighing 20.0–23.0 g were used [Animal Production License No. SCXK (Beijing) 2016–0006]. Animal experiments were conducted in the Animal Room of Tianjin Institute of Medical and Pharmaceutical Science. Rabbits were maintained in conventional animal rooms and individually housed in stainless steel cages, while mice were housed in a cleanroom environment in cages. Animals were maintained in air-conditioned rooms with a temperature of 22 °C ± 3 °C, 55% ± 15% relative humidity, and 12-h light/dark cycle. Animals were fed a commercial diet from Keao Xieli Feed (Beijing, China) twice daily and given tap water (rabbits) or purified water (mice) ad libitum. Standard procedures and housing conditions were applied in a facility accredited by the Tianjin Municipal Science and Technology Bureau. Animals were anesthetized before euthanasia. Permission and approval for animal studies were obtained from the Animal Ethics Committee of Tianjin Institute of Medical & Pharmaceutical Science (Approval No. IMPS-EAEP-Q-2020-01).

### Chemical and reagents

Brimonidine powder (Batch No. AG08-PK20200701) was purchased from Xingkaiyue Biotechnology (Shenzhen, China). Xylazine hydrochloride injection (2 mL/0.2 g, Batch No. 20191002) was purchased from Best Biological Technology Institute (Changsha, China). Chloral hydrate powder (Batch No. 2020304) was purchased from Kermel Chemical Reagent (Tianjin, China). Pentobarbital (Batch No. 127 K1005) was purchased from Huanyu Biotechnology (Beijing, China). Methanol (analytical purity, Batch No.20201001) was purchased from Chemical Reagent wholesale company (Tianjin, China).

Chloral hydrate was prepared as 10% chloral hydrate solution in normal saline. Pentobarbital was prepared as 3 mg/mL and 4 mg/mL solutions in physiological saline. A five-fold gradient brimonidine solution series at concentrations of 0.04, 0.008, and 0.0016 mg/mL was prepared for the writhing test. A 1.2-fold gradient brimonidine solution series at concentrations of 5.5, 6.7, 8.0, and 9.6 mg/mL was prepared to determine the ED_50_ for intraperitoneal anesthesia of mice. For the Brimonidine A group, a 1.1-fold gradient solution series at concentrations of 4.5, 4.9, 5.4, and 6.0 mg/mL was used to determine the ED_50_ for intravenous anesthesia in rabbits. For the Brimonidine B group, a 1.1-fold gradient solution series at concentrations of 37.5, 41.5, 45.5, and 50.0 mg/mL was used to determine the ED_50_ for muscular anesthesia in rabbits. A 0.85-fold gradient series of brimonidine solutions at concentrations of 52.1, 44.2, 37.6, and 32.0 mg/mL was used to determine LD_50_ by intraperitoneal injection in mice. A 0.9-fold gradient series of brimonidine solutions at concentrations of 18.0, 16.4, 14.9, 13.5, and 12.3 mg/mL was used to determine LD_50_ by intravenous injection in rabbits.

To combine brimonidine and chloral hydrate, brimonidine at a dose of 2 mg/mL was added to a 10% solution of chloral hydrate. To combine brimonidine and pentobarbital, brimonidine at a dose of 1 mg/mL was added to a 3 mg/mL solution of pentobarbital.

### Potentiation of pentobarbital sleeping time in mice

Mice were randomly divided into experimental, control, and threshold-dose pentobarbital groups (six mice per group, three male and three female). Each animal in the experimental group was intraperitoneally injected with a combination of brimonidine and pentobarbital at a dose of 0.1 mL/10 g, while the control group and threshold-dose pentobarbital group were intraperitoneally injected with 4 mg/mL and 3 mg/mL pentobarbital solution at a dose of 0.1 mL/10 g, respectively. Mice were then placed in an incubator to maintain their body temperature. Whether the righting response disappeared was recorded, as was the time of disappearance and recovery of the righting response. Onset of sleep was the time that animals stayed immobile and lost their righting reflex.

### Assessment of analgesic activity using an acetic acid-induced writhing test

Mice were randomly divided into high-dose (0.4 mg/kg), medium-dose (0.08 mg/kg), low-dose (0.016 mg/kg), and control groups, with three males and three females per group. Mice in high-, middle-, and low-dose groups were intraperitoneally injected with 0.04, 0.008, and 0.0016 mg/mL brimonidine solution at a dose of 0.1 mL/10 g, respectively, while mice in the control group were intraperitoneally injected with normal saline at a dose of 0.1 mL/10 g. Mice in the control group were intraperitoneally injected with 0.1 mL/10 g saline. Fifteen minutes after administration of all treatments, mice were intraperitoneally injected with 0.6% acetic acid at a dose of 10 mL/kg. Numbers of writhing responses observed during a 20-min period were counted and recorded. Each writhe, defined as the stretching of the abdomen and/or stretching of at least one hind limb, was recorded with a stopwatch.

The percentage of analgesic activity was calculated as follows:

$$\%\mathrm{inhibition}=\left(\mathrm{Wc}-\mathrm{Wt}\right)/\mathrm{Wc}\times 100\%$$

where W is the number of writhings, c is the negative control, and t is the test.

### Determination of hypnotic ED_50_ of intraperitoneal injection in mice

Mice were divided into four groups (five males and five females per group) that were administered 55.6, 66.7, 80.0, or 96.0 mg/kg brimonidine. Mice in each group were intraperitoneally injected with a 1.2-fold gradient brimonidine solution series at a dose of 0.1 mL/10 g. When mice were still in the supine position, they were placed on the heat preservation table in the supine position to maintain their body temperature.

Observation indicators: (1) Behavioral and autonomous activities of mice: the appearance, posture, and mental state of mice were observed as activity (lifting and walking) and inactivity (prostrate without motion). (2) Ratio of sleeping: the incidence of loss of righting response was observed following drug administration. The index of sleep falling was evaluated as the loss of righting response for more than 1 min. Numbers for each group were observed in mice sleeping within 1 h. (3) Sleeping time: the time from administration to the disappearance of righting response and recovery of righting response was recorded. The time from drug administration to disappearance of the righting response was considered the sleep latency, while the time from disappearance of the righting response to recovery of the righting response was considered the sleep duration.

Calculation of ED_50_ by modified Karber method

$${\mathrm{ED}}_{50}={\lg}^{-1}\left[\mathrm{Xm}-\mathrm{I}\left(\Sigma \mathrm{P}-0.5\right)\right]$$

- Xm: logarithmic value of the maximum-dose group dose
- I: Logarithmic ratio of high-dose to low-dose for two adjacent groups
- P: Ratio of sleeping of animals in each group, expressed as a decimal
  $${\displaystyle \begin{array}{l}{\mathrm{ED}}_{50}95\%\mathrm{CI}={\lg}^{-1}\left({\mathrm{lgED}}_{50}\pm 1.96{\mathrm{S}}_{\mathrm{lgED}50}\right)\\ {}{\mathrm{S}}_{\mathrm{lgED}50}=\mathrm{I}\ast {\left[\left(\Sigma \mathrm{P}-{\Sigma \mathrm{P}}^2\right)/\left(\mathrm{n}-1\right)\right]}^{0.5}\end{array}}$$
- n: number of animals in each group

### Determination of hypnotic ED_50_ of intravenous rabbits

Rabbits were divided into four groups (*n* = 10): 4.5 mg/kg, 4.9 mg/kg, 5.4 mg/kg, and 6.0 mg/kg. Rabbits in each group were injected with a 1.1-fold gradient solution series of Brimonidine A at a dose of 1.0 mL/kg. When rabbits were inactive, they were required to assume the supine position.

Observation indexes: (1) Behavioral and autonomic activities of rabbits: the appearance, posture, and mental state of rabbits were characterized by when they walked with or without using their legs (considered as activity), while their prostration was considered as quiet. (2) Ratio of sleeping: the incidence of disappearance of acupuncture response after medication was observed. The index of acupuncture sleep falling was calculated as the disappearance of acupuncture response for more than 1 min, while sleep falling numbers were observed for 1 h in each group. (3) Sleeping time: time from the disappearance of acupuncture response to the recovery of acupuncture response was calculated as the sleeping time.

The modified Karber method was used to calculate ED_50_, as described above.

### Determination of hypnotic ED_50_ of rabbits injected intramuscularly

Rabbits were divided into four groups (*n* = 10 per group): 7.5 mg/kg, 8.3 mg/kg, 9.1 mg/kg, and 10.0 mg/kg. Rabbits in each group were injected with a 1.1-fold gradient solution series of Brimonidine B at a dose of 0.2 mL/kg. When rabbits were inactive, they were required to assume the supine position.

Observational indexes and the calculation method of ED_50_ were identical to those described above for determination of the hypnotic ED_50_ of intravenous injection hypnosis in rabbits.

### Determination of hypnotic ED_50_ of rabbits injected intrarectally

Rabbits were divided into four groups (*n* = 10): 7.5 mg/kg, 8.3 mg/kg, 9.1 mg/kg, and 10.0 mg/kg. Rabbits in each group were injected with a 1.1-fold gradient solution series of Brimonidine B at a dose of 0.2 mL/kg.

Observation indexes and the calculation method of ED_50_ were identical to those described above for the determination of hypnotic ED_50_ of intravenous and intrarectal injection hypnosis in rabbits.

### Measurement of LD_50_ and therapeutic index of mice after intraperitoneal injection using the up-down method

Seventeen KM mice were intraperitoneally injected with a 0.85-fold gradient series of brimonidine solutions at a dose of 0.1 mL/10 g, one at a time. After the mice rested, they were placed in an incubator to maintain their body temperature.

If the first animal survived, the second animal was given a higher dose. However, if the first animal died or was dying, the second animal was administered a lower dose to obtain a total number of experiments **n** and the mortality **p** of each dose.

Calculation of LD_50_:

$${\mathrm{LD}}_{50}={\lg}^{-1}\left[\Sigma \left(\mathbf{nx}\right)/\Sigma \mathbf{n}\right]$$

S_lgLD50_ = d*{Σ[**p**(1-**p**)/(**n**-1)]}^0.5^, where **d** is the log difference between two adjacent doses.

$${\displaystyle \begin{array}{l}{\mathrm{LD}}_{50}95\%\mathrm{CI}={\lg}^{-1}({\mathrm{lgLD}}_{50}\pm 1.96{\mathrm{S}}_{\mathrm{lgLD}50})\\ {}\mathrm{Therapeutic}\ \mathrm{index}={\mathrm{LD}}_{50}/{\mathrm{ED}}_{50}\end{array}}$$

### Measurement of LD_50_ and therapeutic index of rabbits after intravenous injection using the up-down method

Rabbits were intravenously injected with a 0.9-fold gradient series of brimonidine solutions at a dose of 10 mL/1 kg, one at a time. LD_50_ was measured and calculated as described above for the determination of LD_50_ in mice.

### Synergistic effects of brimonidine and chloral hydrate on hypnosis in rabbits

Twenty-four rabbits were divided into three groups (eight rabbits per group): low-dose, high-dose, and control groups. The low-dose group was intravenously injected with 0.3 mL/kg brimonidine and chloral hydrate, while the high-dose group was injected with 0.6 mL/kg brimonidine and chloral hydrate. After intramuscular injection of 0.15 mL/kg xylazine, the control group was injected with 1.5 mL/kg of 10% chloral hydrate at a rate of 2–3 mL/min.

Behavioral and voluntary activities were recorded from the time of administration to the disappearance and recovery of acupuncture responses. Sleep latency was recorded as the time between pentobarbital administration and sleep onset. Sleeping time was observed as described above for the determination of hypnotic ED_50_ in rabbits given intravenous injection. After the acupuncture response recovered, rabbits in low-dose and high-dose groups were intravenously injected with 0.15 mL/kg brimonidine and chloral hydrate, and then the re-sleeping time was observed.

### Systemic absorption

For each rabbit (*n* = 3), 6 mg/mL brimonidine was injected to the ear vein. Blood samples were collected from each rabbit at 5, 15, 30, and 45 min; and 1, 2, and 3 h via the heart. Blood samples were cooled on ice immediately after blood sampling, and plasma was obtained by centrifugation (2000×g for 10 min). All bioanalysis samples were stored at − 80 °C until processing. Brimonidine concentrations in plasma samples were determined by high-performance liquid chromatography (HPLC) assay. The plasma samples were pretreated with methanol extraction methods. The extracted samples were analyzed by HPLC-20A (Shimadzu, Japan). Brimonidine was eluted on a ODS-BP column (4.6 mm*150 mm, 5 μm, Elite, China) using a mobile phase of 15% methanol. Brimonidine was detected at 246 nm. T_1/2_ and area under the curve (AUC_0–∞_) in the plasma were determined.

### Statistical test

One-way ANOVA and Dunnett’s t-test were used to calculate the significance of potentiation of pentobarbital sleeping time and writhing tests in mice, as well as the synergistic effect of brimonidine and chloral hydrate. Statistical analysis was performed by SPSS 17.0 (IBM, Armonk, NY, USA).

## Results

### Brimonidine potentiates pentobarbital hypnosis

**Fig. 1:**
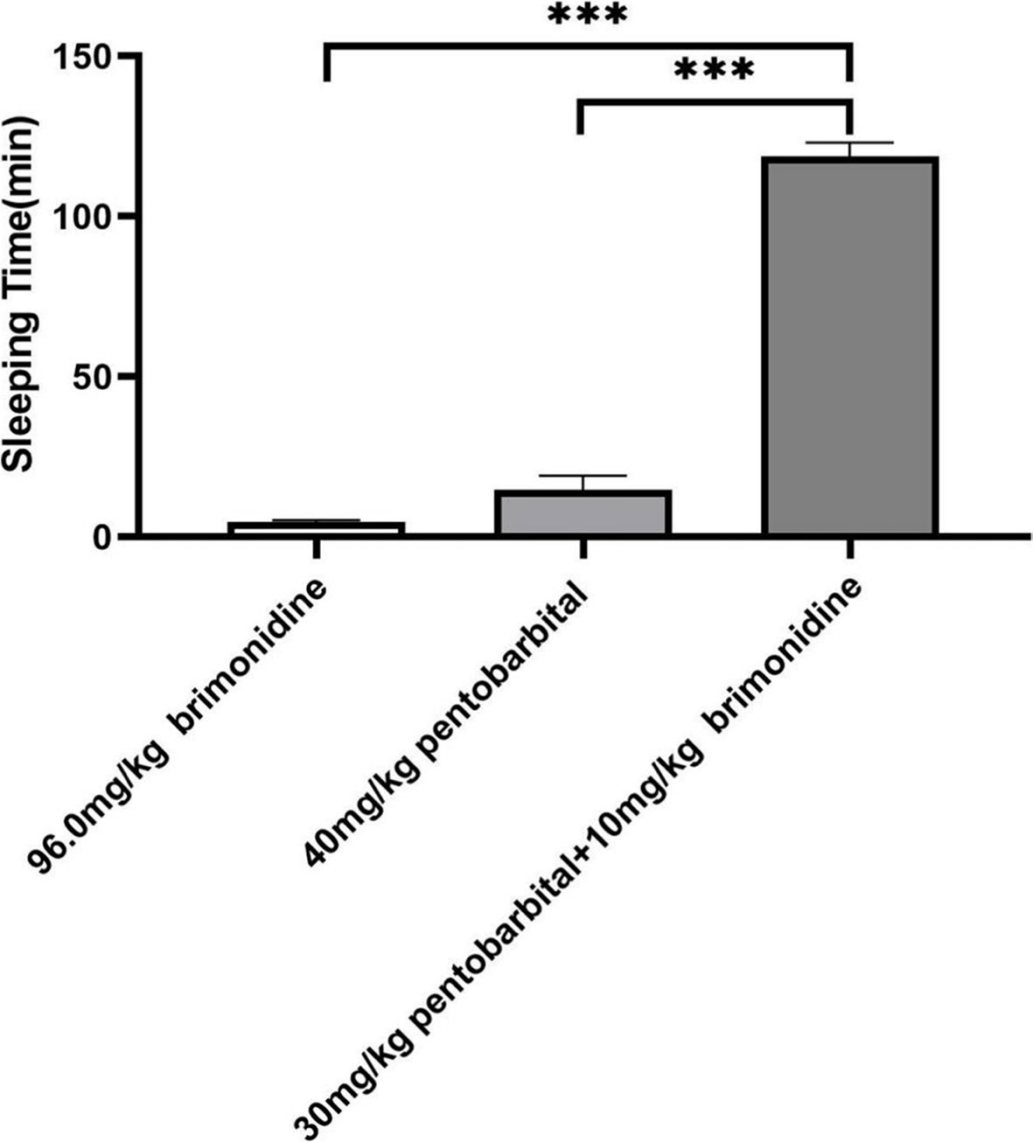
The sleeping time of mice with intraperitoneal injection. Data shown represent mean ± SEM. *** *P* < 0.001(one-way ANOVA, followed by Dunnett’s test)

After injection, mice in the experimental groups gradually developed ataxia and decreased exercise. Approximately 3–5 min after injection, mice exhibited a negative rollover response and the ratio of sleeping was 100%, as defined by an increased sleeping time (118.7 ± 4.6 min) in treated mice compared with the control group (14.8 ± 4.4 min, Fig. 1, Supplement Table 1). Most animals urinated within 0.5 h of administration.

After intraperitoneal injection of pentobarbital in the threshold-dose (30 mg/kg) and control groups (40 mg/kg), mice developed ataxia, decreased their mobility, and then lay down quietly. However, during the 2-h observation period, the ratio of sleeping in the threshold-dose pentobarbital group was always 0, and no micturition occurred.

### Writhing test in mice

Following intraperitoneal injection of brimonidine for 15 min, high-dose group mice exhibited decreased activity. The middle-dose group was still active, but there was no courtship action, whereas the low-dose group moved freely and exhibited courtship actions such as raising forelimbs. In the high-dose group (0.4 mg/kg), there was no writhing response within 20 min of intraperitoneal injection of acetic acid solution. In the middle-dose group (0.08 mg/kg), most mice showed writhing reactions (Supplement Table 2).

In the acetic acid-induced writhing model, the low-dose group exhibited a significant analgesic effect with 82.5% reduction in writhing responses at a dose of 0.016 mg/kg compared with the control group (Table 1). Brimonidine showed a good analgesic effect that was enhanced further at higher doses.

**Table 1:** Analgesic activity of brimonidine assessed by acetic acid-induced writhing in mice

Group	Dose mg/kg	Number of writhes	% Inhibition
Control	0	63.0 ± 7.3	–
High dose	0.4	0***	100
Medium dose	0.08	7.8 ± 4.3***	87.6
Low dose	0.016	10.6 ± 4.5***	82.5

***Compared with control group, *p* < 0.001 (one-way ANOVA, followed by Dunnett’s test)

### Hypnotic ED_50_ of intraperitoneal injection in mice

After injection, mice first exhibited anxiety, followed by ataxia, decreased exercise, slow and deepened breathing, white fundus, and obvious micturition. Moreover, some mice exhibited negative, positive, and negative responses one after another. The ratio of sleeping in the 96 mg/kg group reached 100% (Table 2), but the sleeping time was only 4.8 ± 0.5 min (Supplement Table 3).

**Table 2:** Hypnotic effects of intraperitoneal brimonidine injection in mice

Group	Dose mg/kg	N	Hypnotic number	Ratio of sleeping %
1	55.6	10	0	0
2	66.7	10	2	20
3	80.0	10	6	60
4	96.0	10	10	100

Hypnotic effects are summarized in Table 3. The hypnotic ED_50_ of brimonidine solution was 75.7 mg/kg, and the 95% confidence interval (95% CI) was 70.2–81.6 mg/kg.

**Table 3:** Hypnotic effects of intravenous brimonidine in rabbits

Group	Dose mg/kg	N	Hypnotic number	Ratio of sleeping%
1	4.5	10	0	0
2	4.9	10	3	30
3	5.4	10	7	70
4	6.0	10	10	100

### Hypnotic ED_50_ of intravenous injection in rabbits

During half injection, rabbits struggled impatiently for several seconds and then calmed down. During the incubation period, the front legs were straight, the body was shaking, the tail was straight, the pupils became narrowed, the fundus was pale, and the limbs gradually relaxed. During sleep, the rabbit may experience phenomena such as eyeball shaking, micturition, and body surface temperature drop, or may wake up temporarily by changing the body position or twisting the limbs.

Hypnotic effects were summarized in Table 3. The hypnotic ED_50_ of brimonidine solution was 5.2 mg/kg and the 95% CI was 5.0–5.4 mg/kg.

**Fig. 2:**
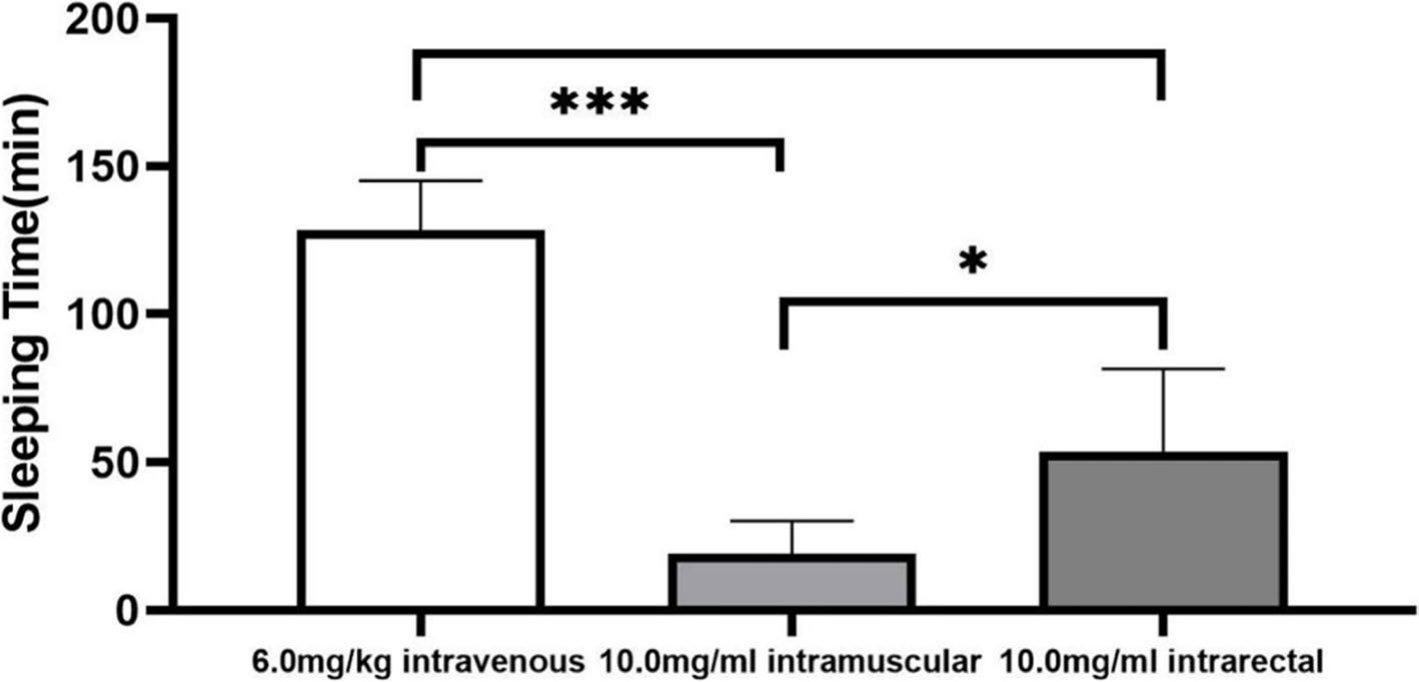
Sleeping times of rabbits following intravenous (6.0 mg/kg), intramuscular (10.0 mg/kg), or intrarectal (10.0 mg/kg) brimonidine injection. Data represent mean ± SEM.* *P* < 0.05; *** P < 0.001 (one-way ANOVA, followed by Dunnett’s test)

In the 6-mg/kg dose group, all rabbits entered the sleep stage, the induction period was 8.2 ± 3.3 min, and sleep duration was 128.1 ± 16.9 min (Fig. 2, Supplement Table 4). However, during the induction period, two rabbits exhibited obvious extrapyramidal reactions (e.g., arch inversion, head and neck elevation, and muscle tremor) and sleeping times of only 6 and 9 min, which were significantly lower than other rabbits in the same group. To verify whether the sleeping time of these two rabbits was shortened due to over-anesthesia, 4.9 mg/kg brimonidine solution was intravenously injected into rabbits 3 days later. Subsequently, no obvious extrapyramidal reaction occurred during the induction period and sleeping times were prolonged to 46 min and 90 min, respectively.

### Hypnotic ED_50_ of intramuscular injection in rabbits

There was no restlessness or struggle during intramuscular injection of rabbits. Moreover, the latency response was obviously reduced compared with intravenous injection, the sleep induction period was less than 3 min, and most rabbits did not exhibit muscle tremor or horn arch rebound. During sleep, rabbits exhibited symptoms such as nystagmus, micturition, and temperature drop on the body surface. Changing the body position or twisting the limbs could temporarily wake up the rabbits.

Hypnotic effects are summarized in Table 4. The hypnotic ED_50_ of intramuscular brimonidine injection was 8.8 mg/kg and the 95% CI was 8.5–9.1 mg/kg.

**Table 4:** Hypnotic effects of intramuscular brimonidine in rabbits

Group	Dose mg/kg	N	Hypnotic number	Ratio of sleeping %
1	7.5	10	0	0
2	8.3	10	2	20
3	9.1	10	7	70
4	10.0	10	10	100

All rabbits in the 10-mg/kg group fell asleep, with an induction period of 6.4 ± 1.8 min and sleeping time of 19.0 ± 11.3 min (Fig. 2, Supplement Table 5).

### Hypnotic ED_50_ of intrarectal injection in rabbits

There was no restlessness or struggle during intrarectal injection of rabbits. Neither muscle tremors nor the horn arch rebound was observed.

Hypnotic effects are summarized in Table 5. The hypnotic ED_50_ of intrarectal brimonidine injection was 8.7 mg/kg and the 95% CI was 8.3–9.1 mg/kg.

**Table 5:** Hypnotic effects of intrarectal brimonidine in rabbits

Group	Dose mg/kg	N	Hypnotic number	Ratio of sleeping %
1	7.5	10	1	10
2	8.3	10	3	30
3	9.1	10	6	60
4	10.0	10	10	100

All rabbits in the 10-mg/kg group fell asleep, with an induction period of 11.0 ± 4.1 min and sleeping time of 53.6 ± 27.9 min (Fig. 2, Supplement Table 6).

### LD_50_ of mice was determined with an up-and-down sequence

The survival and death of mice after intraperitoneal brimonidine injection were evaluated with an up-and-down sequence. The results are summarized in Table 6 and Supplement Table 7.

**Table 6:**
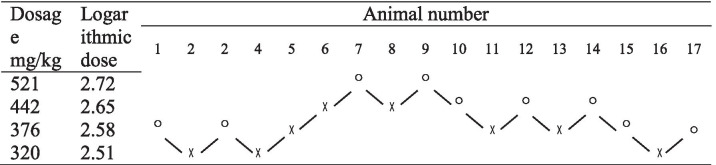
Survival and death of mice after intraperitoneal brimonidine injection

◯ Mouse died after injection, ╳ Mouse survived after injection

The LD_50_ of intraperitoneal brimonidine injection in mice was calculated to be 379 mg/kg, with a 95% CI of 343–420 mg/kg and therapeutic index of 5.0.

### LD_50_ of mice was determined with an up-and-down sequence

Survival and death of rabbits after intraperitoneal brimonidine injection are summarized in Table 7 and Supplement Table 8.

**Table 7:**
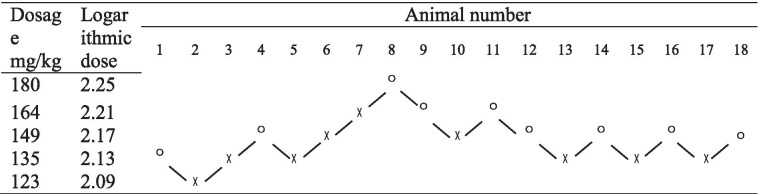
Survival and death of rabbits after intravenous brimonidine injection

◯ Rabbit died after injection, ╳ Rabbit survived after injection

The LD_50_ of intravenous brimonidine injection in rabbits was calculated to be 146 mg/kg, with a 95% CI of 135–157 mg/kg and therapeutic index of 28.0.

### Synergistic effects of brimonidine and chloral hydrate

**Fig. 3:**
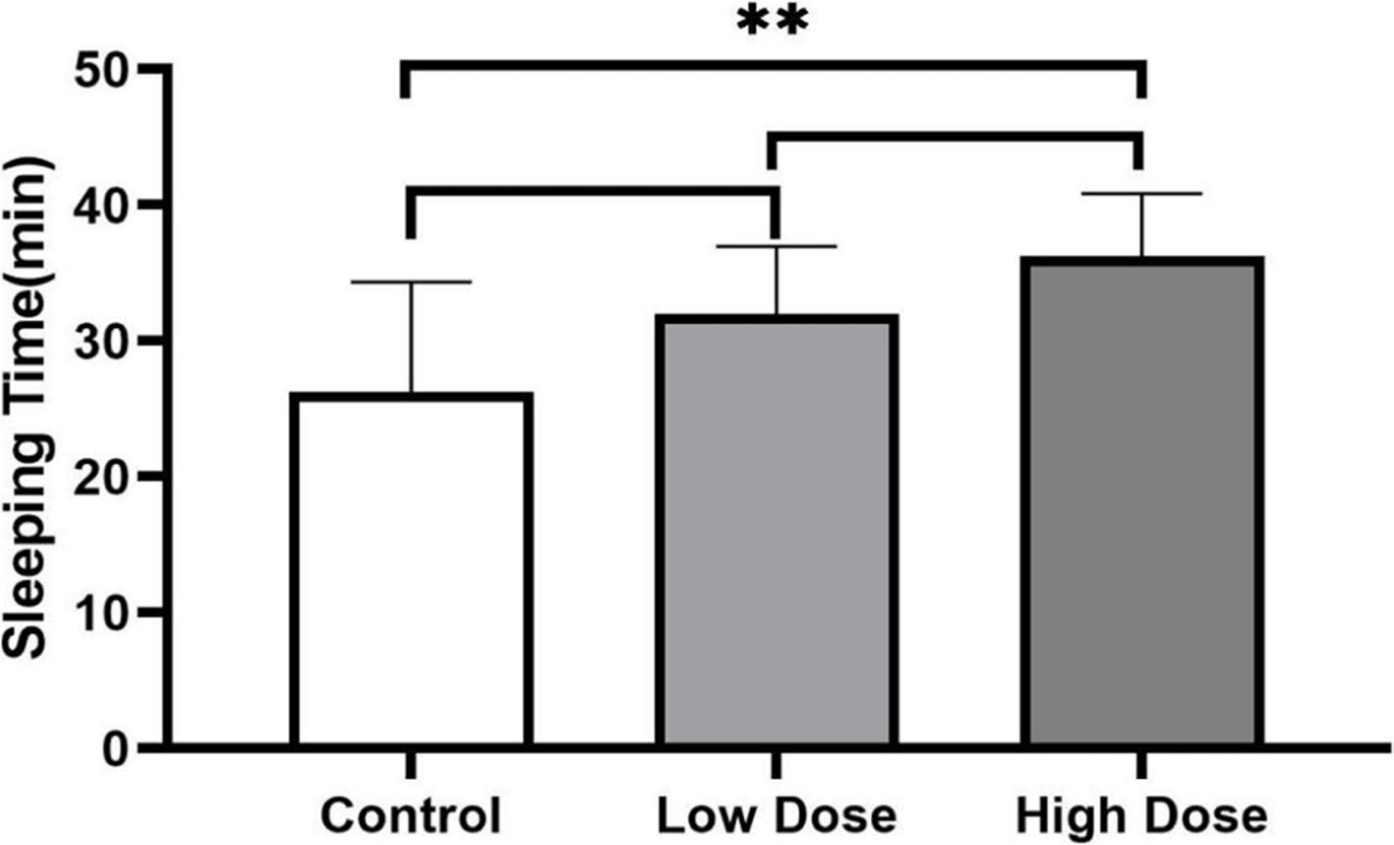
Sleeping times of rabbits in the control group (0.15 mg/kg xylazine + 0.15 mg/kg chloral hydrate), low-dose group (0.6 mg/kg brimonidine + 0.03 mg/kg chloral hydrate), and high-dose group (1.2 mg/kg brimonidine + 0.06 mg/kg chloral hydrate). ** *P* < 0.01 (one-way ANOVA, followed by Dunnett’s test)

The induction periods of low-dose and high-dose groups were shorter than 1 min. After intravenous injection, all animals exhibited miosis, white fundus, turning over, decreased muscle tension, and no limb stiffness. Rabbits in both groups entered the anesthesia period with similar sleeping times. During the anesthesia period, rabbits exhibited nystagmus and decreased body surface temperatures, but did not urinate and could not temporarily wake up by changing the body position or twisting the limbs. In the control group, the induction period was 3.3 ± 1.4 min, mild symptoms (such as limb stiffness) were exhibited, and one death occurred (Supplement Table 9). Sleeping times of control group animals were shorter than observed in high-dose group animals (Fig. 3; *P* < 0.01). However, there was no difference in sleeping times between low-dose and high-dose groups (Fig. 3; *P* > 0.05).

### Systemic absorption

**Fig. 4:**
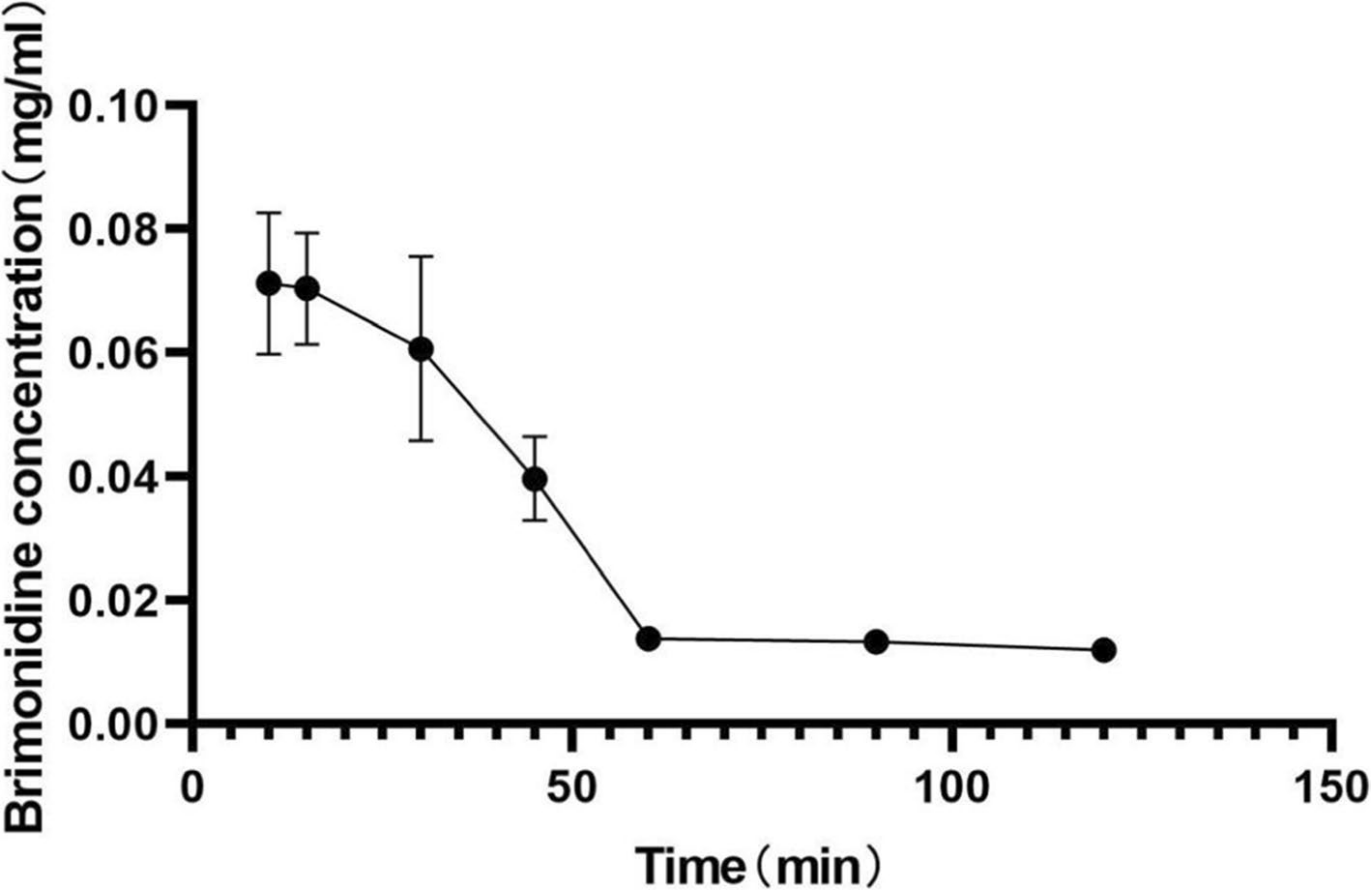
Brimonidine concentrations in the plasma after the intravenous administration. Data are presented as mean ± standard deviation (*n* = 3)

The brimonidine plasma concentrations in the intravenous (6.0 mg/kg) rabbits which are exhibited in Fig. 4 and Supplement Table 10 can be described by a first-order elimination, single-compartment pharmacokinetic model. T_1/2_ values were 34.7 min. AUC_0–∞_ values were 3.902 mg/ml·min.

## Discussion

Brimonidine is clinically used in the treatment of glaucoma and facial congestion. No previous study has investigated the induction or maintenance of general anesthesia with brimonidine; however, a report of brimonidine is used for pre-anesthetic administration for animals, such as intraperitoneal injection of 30 μg/kg brimonidine, diminished the sympathetic nerve effect of ketamine general anesthesia in rats [1].

Many clinical reports indicate that local and systemic administration of brimonidine can cause side effects in the central nervous system, resulting in nervous system symptoms such as sedation, lethargy, and even death [2]. Following intravenous injection of 1 mg/kg brimonidine, the rats stopped moving, their pupils dilated (different from those of primates and rabbits, which may be related to species specificity), and they calmed down. After intravenous injection of 2 mg/kg brimonidine, the rats breathed slowly and quietly, and the dose did not reach the level of subliminal anesthesia [3]. Toxicity testing revealed that a large dose (5 mg/kg) of oral brimonidine elicited short-term hypnotic and sedative effects in rabbits, which also exhibited disappearance of nerve responses and reduced muscle tension [4]; however, the effect of general anesthesia was not studied in depth.

This study confirmed that brimonidine has good hypnotic, sedative, and analgesic effects on mice and rabbits. This result is related to the α_2_ receptor agonist action of brimonidine [5].

Brimonidine is highly receptor-selective and has more advantages than dexmedetomidine and clonidine. The α_2_:α_1_ selectivity coefficient of brimonidine is 1780:1, which is higher than 1620:1 for dexmedetomidine and 220:1 for clonidine [6]. In addition, the α_2_:I_1_ selectivity coefficient of brimonidine is 100:1, which is higher than 32:1 for dexmedetomidine and 16:1 for clonidine [7]. There are three α_2_ receptor subtypes in humans, primates, pigs, and rabbits (α_2a_, α_2b_, and α_2c_), of which the α_2a_ receptor is involved in general anesthesia. Brimonidine has moderate α_2a_ receptor selectivity for α_2_ receptor subtypes. The K_i_ values of dexmedetomidine for α_2a_, α_2b_, and α_2c_ receptors are 6.2, 4.0, and 6.0 nM, respectively, whereas these values are 3.7, 512, and 120 nM, respectively, for brimonidine [8]. Activation of α_1_, I_1_, and α_2b_ receptors affects the stability of cardiovascular dynamics [7]. Due to its reduced affinity for α_1_, I_1_, and α_2b_ receptors, the side effects of brimonidine are lighter than clonidine and dexmedetomidine [9].

The locus coeruleus brainstem is an important functional area in the central nervous system that regulates arousal. Activation of α_2a_ receptors hyperpolarizes nerve cells in the locus coeruleus, which reduces the release of norepinephrine and inhibition of γ-GABA neurons in the ventrolateral anterior cross nucleus of the hypothalamus to induce sedative hypnotic effects. The ED_50_ of brimonidine for inducing potassium influx in rat locus coeruleus neurons in vitro is 4.6 * 10^− 8^ M, while 10^− 6^ M brimonidine can induce the maximum current [10]. The hypnotic mechanism of brimonidine is similar to that observed in normal non-eye-moving sleep. During observations of the hypnosis of rabbits, we found that the eyeball moves back and forth rapidly and slightly, and it is speculated that the rabbits entered a state similar to sleep.

The hypnotic assessment method was based on prolongation of sleep induced by pentobarbital, a classic pharmacological method for screening sedative hypnotic agents [11, 12]. The hypnotic effect was evaluated by sleep time duration induced by a subhypnotic dose of pentobarbitone. Brimonidine showed hypnotic activity that increased sleeping time induced by pentobarbitone.

In this study, we determined ED_50_ values for mouse hypnosis (78 mg/kg for intraperitoneal administration) and rabbit hypnosis (5.20 mg/kg for intravenous injection, 8.8 mg/kg for intramuscular injection, and 8.7 mg/kg for intrarectal injection), as well as sleeping times of mice (4.8 ± 0.5 min for intraperitoneal injection) and rabbits (128.1 ± 16.9 min for intravenous injection, 19.0 ± 11.3 min for intramuscular injection, and 53.6 ± 27.9 min for intrarectal injection). The observed difference in hypnotic doses of brimonidine for mice and rabbits may be related to the difference in species. Thus, if brimonidine is developed as an anesthetic for experimental animals, it is more suitable to be used alone in rabbits than mice.

The α_2_ receptor agonist can exert analgesic and noxious stimulation inhibitory effects in peripheral nerves, spinal cord, and upper spinal cord [13]. The α_2a_ subtype is contained in the spinal dorsal horn and primary sensory neurons, whereby adrenergic receptor agonists bind this receptor to trigger postsynaptic inhibition of secondary afferent neurons and presynaptic inhibition of primary sensory neurons, thus playing a major role in inhibiting pain signals [14].

The acetic acid-induced writhing reflex mouse model is a widely accepted, simple, sensitive, and effective pain model for evaluating peripherally acting analgesics. The pain caused by acetic acid liberates endogenous substance to elicit peripheral- and central-mediated analgesic actions [15]. In the writhing test of mice, the analgesic effect of brimonidine at a dose of 0.016 mg/kg was obvious—this dose is 4800 times lower than the hypnotic ED_50_. Compared with 0.016 mg/kg brimonidine, 0.08 mg/kg brimonidine had a sedative effect and an increased analgesic effect. A dose of 0.4 mg/kg brimonidine decreased the activity of mice, and no acetic acid-induced peritoneal pain reaction was observed. These results indicate that brimonidine significantly reduced the number of writhes in a dose-dependent manner compared with the negative control. However, because sedative and analgesic effects are sometimes difficult to distinguish, especially for elevated concentrations, it remains uncertain whether the disappearance of pain responses in high-dose group mice was entirely due to the analgesic activity of the drug or partly affected by sedation.

Previous studies reported that brimonidine had good independent analgesic effects. For example, inhibition of thermal nociception in mice following epidural administration of brimonidine had an analgesic ED_50_ of 0.37 nM, and the combination of brimonidine with an epidural (i.e. opioid) led to synergistic analgesia [16].

The hypnotic effect of brimonidine was more obvious in rabbits than in mice. Our experiments found that intravenous administration of 4.5 mg/mL brimonidine could make the righting response disappear. During this period, the limbs could be placed at will, but pinching the limbs could cause muscles of the limbs to contract. Although α_2_ receptor agonists can theoretically cause presynaptic inhibition of motor neurons and play a certain role in muscle relaxation [17], muscle relaxation is mainly caused by higher central inhibition. The limb muscle contraction caused by the clamp proves that the sedative effect of the above dose was good, but it did not achieve a hypnotic effect. The subthreshold dose of brimonidine in rabbits was far less than the anesthetic effective dose (not obvious in mice), but a good sedative effect at the low dose still caused some interference in hypnotic dose experiments in rabbits. Therefore, in the hypnotic half-effective-amount experiment of rabbits under intravenous intramuscular anesthesia, the acupuncture response rather than the righting response was specifically selected as the test criterion for sleep. The dose of brimonidine that inhibited the acupuncture response maintained the depth of anesthesia for simple surgery in small animals, while the low dose of brimonidine was suitable for pre-physical sedation.

Although brimonidine has hypnotic and analgesic effects because it indirectly inhibits the ascending activation system of the reticular structure through the locus coeruleus, it does not block the awakening mechanism while accompanying sleep [18]. We found that during the hypnotic process elicited by injected administration of brimonidine to rabbits, they would be awakened in a short time by changing their body position and pulling their limbs hard. Therefore, brimonidine alone is only suitable for minor operations that do not involve frequent physical renewal. In addition, animals urinated during anesthesia, which might be due to the high dose of brimonidine acting on α_2_ receptors in the paraventricular nucleus of the hypothalamus, thereby inhibiting the release of antidiuretic hormone to cause an increase in urine output. Simultaneously, brimonidine inhibited norepinephrine release and contraction of the bladder sphincter, leading animals to urinate. Dexmedetomidine had a similar effect on diuresis in clinical applications [19]. This is disadvantageous for simple anesthesia of animals without catheter insertion because sudden micturition can affect disinfection of the surgical area.

After intravenous administration of a high dose of brimonidine, the hypnotic effect was weakened, not enhanced. In previous experiments, a large portion of animals may not have been fully anesthetized when the intravenous drug dose was more than 6.7 mg/kg or intrarectal dose was more than 12.3 mg/kg. During induction of general anesthesia, high-dose α_2_ receptor agonists can activate dopaminergic neurons in the ventral tegmental area, which increases dopamine concentrations in the related forebrain projection areas [20]. During anesthesia, extrapyramidal symptoms appeared in some rabbits, which might further weaken the inhibitory effect of drugs on central adrenal energy and/or alter the anesthetic effect.

When judging the anesthetic effect of brimonidine alone or in combination with chloral hydrate by various routes of administration, the recommended dose-selection principle (6 mg/kg for intravenous injection, 10 mg/kg for intramuscular injection or intrarectal injection) is to use the minimum amount of drug that achieves a sufficient anesthetic effect in all animals with minimal side effects.

To overcome these shortcomings and solve potential problems with future application, the combination of brimonidine with other anesthetics is a good method. The results of experiments examining the hypnotic effect of brimonidine on increasing the threshold dose of pentobarbital confirmed the hypnotic effect of brimonidine. Moreover, these results revealed that 10 mg/kg brimonidine combined with 30 mg/kg pentobarbital hypnotized all mice for more than 2 h after intraperitoneal injection, without any death phenomenon, which is beneficial for long-term operations of mice. However, α_2_ receptor agonists such as brimonidine can lead to hypothermia [21], meaning small animals such as mice and rats may be injured or die. Therefore, in this study, mice were incubated to maintain a normal physiological body temperature.

In addition to pentobarbital, a combination of brimonidine and chloral hydrate was also found to have a good synergistic effect. When brimonidine and chloral hydrate were used together, the doses of the two drugs in the low-dose group were only one-tenth of the original single dose. In addition, the low-dose group exhibited rapid onset of anesthesia, a stable anesthesia effect, and convenient supplement of anesthetics. Although the dosage of the high-dose group was double that of the low-dose group, the anesthetic effect was similar. Thus, combining brimonidine and chloral hydrate has the potential advantages of stable effects and high cost performance for animal anesthesia.

In terms of safety, the therapeutic index of intraperitoneal brimonidine administration in mice was 5.0, while the therapeutic index of intravenous brimonidine in rabbits was 28.0, indicating that the safety range of brimonidine was wider in rabbits than mice. In this study, no mortality occurred during experiments using the ED_50_ of brimonidine. Moreover, no deaths occurred when brimonidine was administered in combination with pentobarbital or chloral hydrate.

Many reports have indicated the safety of brimonidine administered to animals at high doses; for example, oral administration of brimonidine to rats for up to 1 year (2.5 mg/kg/day) did not cause organ toxicity [3]. In a study of reproductive toxicity, oral administration of 5 mg/kg brimonidine to pregnant rabbits caused abortion in individual rabbits because of uterine contraction, and had no teratogenic effect on offspring [4]. According to published pharmacokinetic studies [22], brimonidine is metabolized in the liver and excreted in urine, with a half-life for blood elimination of 3 h and binding rate of 29% for plasma protein. Such experimental conclusions have reference value for the use of brimonidine. This study supports that plasma brimonidine concentrations in rabbits following intravenous administration can be described by a first-order elimination, single-compartment pharmacokinetic model.

Sleeping times of rabbits in the 10-mg/kg intramuscular anesthesia group were shorter than observed in the 10-mg/kg intrarectal anesthesia group (Fig. 2; *P* < 0.05). Use of brimonidine in clinical eye drops as a drug for glaucoma yielded high local absorption without local tissue irritation because of its physical properties. First, the pK_a_ value of brimonidine is 7.78, yielding an aqueous solution with an approximately neutral pH. Second, the logP value of brimonidine is 0.83, which is similar to the logP of clonidine (0.73) used for mucosally administered general sedation [23]. In contrast, the logP value of dexmedetomidine, which exhibits high permeability and low solubility that limit bioavailability for local administration, is 3.44 [24].

In future research, the safety of brimonidine for general anesthesia should be further investigated, such as monitoring of vital signs during anesthesia, investigating possible impacts on blood biochemistry and other indicators, and further confirming the efficacy of brimonidine combined with other anesthetics.

## Conclusions

In summary, brimonidine elicits good sedative and analgesic effects, and is stable and safe; thus, brimonidine has the potential for use as a new general anesthesia drug with multiple routes of administration. The combined anesthesia method of brimonidine and chloral hydrate has the advantages of convenient operation, short induction time, long maintenance time, good analgesic and muscle relaxation effects, low anesthetic dosage, low anesthetic adverse reaction, stable anesthetic effects, high cost performance, and reliable sources. The use of brimonidine alone or in combination with other anesthetics may be a simple, rapid, safe, and effective method to induce sedation and anesthesia in animal experiments and even clinical surgery.

## Supplementary Information

**Additional file 1: Table 1.** Sleeping time (min) of brimonidine potentiates pentobarbital hypnosis in mice.

**Additional file 2: Table 2.** Number of writhes induced by acetic acid in mice.

**Additional file 3: Table 3.** Hypnotic effects of intraperitoneal brimonidine in mice.

**Additional file 4: Table 4.** Hypnotic effects of intravenous brimonidine in rabbits.

**Additional file 5: Table 5.** Hypnotic effects of intramuscular brimonidine in rabbits.

**Additional file 6: Table 6.** Hypnotic effects of intrarectal brimonidine in rabbits.

**Additional file 7: Table 7.** LD_50_ of brimonidine in mice evaluated with up-and-down sequential method.

**Additional file 8: Table 8.** LD_50_ of brimonidine in rabbits evaluated with up-and-down sequential method.

**Additional file 9: Table 9.** Synergy of hypnotic effects of brimonidine combined with chloral hydrate.

**Additional file 10: Table 10.** Brimonidine concentrations in the plasma after the intravenous administration (mg/ml).

## Data Availability

All data generated or analyzed during this study are included in this published article [and its supplementary information files].

## Abbreviations

ED_50_: The median effective dose
LD_50_: The median lethal dose
CI: Confidence interval
logP: Oil - water partition coefficient
P: The ratio of the concentration of the compound in positive alcohol to water
