# Supplementary material for: Preliminary evaluation of the efficacy and safety of brimonidine for general anesthesia
Source: BMC Anesthesiol. 2021 Dec 3;21:305. doi: 10.1186/s12871-021-01516-1 (PMC8641169; doi:10.1186/s12871-021-01516-1)
Supplement: Supplementary file 1 — Additional file 1: Table 1. Sleeping time (min) of brimonidine potentiates pentobarbital hypnosis in mice. [file 12871_2021_1516_MOESM1_ESM.docx]

**Additional file 1**

Table 1 Sleeping time (min) of brimonidine potentiates pentobarbital hypnosis in mice

| Number | Test group | Control group | Threshold-dose group |
| --- | --- | --- | --- |
| 1 | 126 | 10 | 0 |
| 2 | 117 | 15 | 0 |
| 3 | 122 | 13 | 0 |
| 4 | 117 | 14 | 0 |
| 5 | 116 | 14 | 0 |
| 6 | 114 | 23 | 0 |
| $\overline{\text{x}}\pm\text{s}$ | 118.7±4.6 | 14.8±4.4 | 0 |
