# Supplementary material for: Preliminary evaluation of the efficacy and safety of brimonidine for general anesthesia
Source: BMC Anesthesiol. 2021 Dec 3;21:305. doi: 10.1186/s12871-021-01516-1 (PMC8641169; doi:10.1186/s12871-021-01516-1)
Supplement: Supplementary file 2 — Additional file 2: Table 2. Number of writhes induced by acetic acid in mice. [file 12871_2021_1516_MOESM2_ESM.docx]

**Additional file 2**

Table 2 Number of writhes induced by acetic acid in mice

|  | Control | High dose | Medium dose | Low dose |
| --- | --- | --- | --- | --- |
| 1 | 57 | 0 | 10 | 6 |
| 2 | 68 | 0 | 8 | 14 |
| 3 | 73 | 0 | 8 | 8 |
| 4 | 53 | 0 | 8 | 9 |
| 5 | 65 | 0 | 13 | 9 |
| 6 | 62 | 0 | 0 | 18 |
| $\overline{\text{x}}\pm\text{s}$ | 63.0±7.3 | 0 | 7.8±4.3 | 10.6±4.5 |
