# Supplementary material for: Preliminary evaluation of the efficacy and safety of brimonidine for general anesthesia
Source: BMC Anesthesiol. 2021 Dec 3;21:305. doi: 10.1186/s12871-021-01516-1 (PMC8641169; doi:10.1186/s12871-021-01516-1)
Supplement: Supplementary file 3 — Additional file 3: Table 3. Hypnotic effects of intraperitoneal brimonidine in mice. [file 12871_2021_1516_MOESM3_ESM.docx]

**Additional file 3**

Table 3 Hypnotic effects of intraperitoneal brimonidine in mice

|  | 55.6mg/kg | 66.7mg/kg | 80.0mg/kg | 96.0mg/kg |
| --- | --- | --- | --- | --- |
| 1 | NO | NO | 14(5） | 5(11） |
| 2 | NO | NO | 9（10） | 6(9） |
| 3 | NO | NO | NO | 5(11） |
| 4 | NO | NO | 4(15） | 6(20） |
| 5 | NO | 5(20） | 3(12） | 5(8） |
| 6 | NO | NO | 4(4） | 6(9） |
| 7 | NO | NO | 13(6） | 5.5(15） |
| 8 | NO | 4(8） | 5(8） | 5.5(11） |
| 9 | NO | NO | 5(7） | 5(16） |
| 10 | NO | NO | NO | 6(16） |

a (b): Sleeping time (Induction time); NO: Acupuncture reflex was positive during the observation period of 2 h; * indicates abnormal values.
