# Supplementary material for: Preliminary evaluation of the efficacy and safety of brimonidine for general anesthesia
Source: BMC Anesthesiol. 2021 Dec 3;21:305. doi: 10.1186/s12871-021-01516-1 (PMC8641169; doi:10.1186/s12871-021-01516-1)
Supplement: Supplementary file 4 — Additional file 4: Table 4. Hypnotic effects of intravenous brimonidine in rabbits. [file 12871_2021_1516_MOESM4_ESM.docx]

**Additional file 4**

Table 4 Hypnotic effects of intravenous brimonidine in rabbits

|  | 4.5mg/kg | 4.9mg/kg | 5.4mg/kg | 6.0mg/kg |
| --- | --- | --- | --- | --- |
| 1 | NO | NO | 22(11） | 122(13） |
| 2 | NO | NO | NO | 122(9） |
| 3 | NO | NO | NO | 120(8） |
| 4 | NO | 90(6) | 21(9） | 6(12）* |
| 5 | NO | 16(10） | 92(7） | 159(4） |
| 6 | NO | NO | 44(4） | 148(8） |
| 7 | NO | NO | 13(8） | 128(6） |
| 8 | NO | 46(8） | 25(7） | 120(4） |
| 9 | NO | NO | 59(8） | 9(12）* |
| 10 | NO | NO | NO | 108(6） |

a (b): Sleeping time (Induction time); NO: Acupuncture reflex was positive during the observation period of 2 h; * indicates abnormal values.
