# Supplementary material for: Preliminary evaluation of the efficacy and safety of brimonidine for general anesthesia
Source: BMC Anesthesiol. 2021 Dec 3;21:305. doi: 10.1186/s12871-021-01516-1 (PMC8641169; doi:10.1186/s12871-021-01516-1)
Supplement: Supplementary file 5 — Additional file 5: Table 5. Hypnotic effects of intramuscular brimonidine in rabbits. [file 12871_2021_1516_MOESM5_ESM.docx]

**Additional file 5**

Table 5 Hypnotic effects of intramuscular brimonidine in rabbits

|  | 7.5mg/kg | 8.3mg/kg | 9.1mg/kg | 10.0mg/kg |
| --- | --- | --- | --- | --- |
| 1 | NO | NO | 54(4） | 29(8） |
| 2 | NO | NO | 37(4） | 39(8） |
| 3 | NO | 37(4) | NO | 5(8） |
| 4 | NO | NO | 33(4） | 13(8） |
| 5 | NO | NO | 16(4） | 17(8） |
| 6 | NO | NO | 53(6） | 31(6） |
| 7 | NO | NO | NO | 9(6） |
| 8 | NO | 14(5） | 38(5） | 25(4） |
| 9 | NO | NO | NO | 12(4） |
| 10 | NO | NO | 13(6) | 10(6） |

a (b): Sleeping time (Induction time); NO: Acupuncture reflex was positive during the observation period of 2 h.
