# Supplementary material for: Preliminary evaluation of the efficacy and safety of brimonidine for general anesthesia
Source: BMC Anesthesiol. 2021 Dec 3;21:305. doi: 10.1186/s12871-021-01516-1 (PMC8641169; doi:10.1186/s12871-021-01516-1)
Supplement: Supplementary file 6 — Additional file 6: Table 6. Hypnotic effects of intrarectal brimonidine in rabbits. [file 12871_2021_1516_MOESM6_ESM.docx]

**Additional file 6**

Table 6 Hypnotic effects of intrarectal brimonidine in rabbits

|  | 7.5mg/kg | 8.3mg/kg | 9.1mg/kg | 10.0mg/kg |
| --- | --- | --- | --- | --- |
| 1 | 27(17） | 27(22） | 21(25） | 48(18） |
| 2 | NO | NO | 49(13） | 37(9） |
| 3 | NO | NO | NO | 42(18） |
| 4 | NO | NO | NO | 34(13） |
| 5 | NO | 72(12） | 26(13） | 55(9） |
| 6 | NO | 13(9） | 28(10） | 42(11） |
| 7 | NO | NO | 41(12） | 71(8） |
| 8 | NO | NO | 100(11） | 39(10） |
| 9 | NO | NO | NO | 41(6） |
| 10 | NO | NO | NO | 127(8） |

a (b): Sleeping time (Induction time); NO: Acupuncture reflex was positive during the observation period of 2 h.
