# Supplementary material for: Preliminary evaluation of the efficacy and safety of brimonidine for general anesthesia
Source: BMC Anesthesiol. 2021 Dec 3;21:305. doi: 10.1186/s12871-021-01516-1 (PMC8641169; doi:10.1186/s12871-021-01516-1)
Supplement: Supplementary file 7 — Additional file 7: Table 7. LD50 of brimonidine in mice evaluated with up-and-down sequential method. [file 12871_2021_1516_MOESM7_ESM.docx]

**Additional file 7**

Table 7 LD_50_ of brimonidine in mice evaluated with up-and-down sequential method

| Logarithmic dose  x | mortality  r | Survival  s | Total  n | mortality rate  p | nx | p(1-p)/(n-1) |
| --- | --- | --- | --- | --- | --- | --- |
| 2.72 | 2 | 0 | 2 | 0.0000 | 5.38 | 0 |
| 2.65 | 3 | 2 | 5 | 0.6000 | 13.20 | 0.0600 |
| 2.58 | 4 | 3 | 7 | 0.5714 | 17.92 | 0.0408 |
| 2.51 | 0 | 3 | 3 | 1.0000 | 7.35 | 0 |
| add up to | 9 | 8 | 17 | - | 43.85 | 0.1008 |
