# Supplementary material for: Preliminary evaluation of the efficacy and safety of brimonidine for general anesthesia
Source: BMC Anesthesiol. 2021 Dec 3;21:305. doi: 10.1186/s12871-021-01516-1 (PMC8641169; doi:10.1186/s12871-021-01516-1)
Supplement: Supplementary file 8 — Additional file 8: Table 8. LD50 of brimonidine in rabbits evaluated with up-and-down sequential method. [file 12871_2021_1516_MOESM8_ESM.docx]

**Additional file 8**

Table 8 LD_50_ of brimonidine in rabbits evaluated with up-and-down sequential method

| Logarithmic dose  x | mortality  r | Survival  s | Total  n | mortality rate  p | nx | p(1-p)/(n-1) |
| --- | --- | --- | --- | --- | --- | --- |
| 2.25 | 1 | 0 | 1 | 0.0000 | 2.26 | 0 |
| 2.21 | 2 | 1 | 3 | 0.6667 | 6.63 | 0.1111 |
| 2.17 | 5 | 2 | 7 | 0.7143 | 15.19 | 0.0340 |
| 2.13 | 1 | 5 | 6 | 0.1667 | 12.78 | 0.0278 |
| 2.09 | 0 | 1 | 1 | 1.0000 | 2.09 | 0 |
| add up to | 9 | 8 | 18 | - | 38.95 | 0.1729 |
