# Supplementary material for: Preliminary evaluation of the efficacy and safety of brimonidine for general anesthesia
Source: BMC Anesthesiol. 2021 Dec 3;21:305. doi: 10.1186/s12871-021-01516-1 (PMC8641169; doi:10.1186/s12871-021-01516-1)
Supplement: Supplementary file 9 — Additional file 9: Table 9. Synergy of hypnotic effects of brimonidine combined with chloral hydrate. [file 12871_2021_1516_MOESM9_ESM.docx]

**Additional file 9**

Table 9 Synergy of hypnotic effects of brimonidine combined with chloral hydrate

|  | Control | Low dose | | High dose | |
| --- | --- | --- | --- | --- | --- |
|  | The first  administration | The first  administration | The second administration* | The first  administration | The second administration* |
| 1 | 23(3） | 32(1） | 35(1） | 31(1） | 30(1） |
| 2 | 39(6） | 21(1） | 23(1） | 29(1） | 24(1） |
| 3 | Dead | 36(1） | 32(1） | 37(1） | 25(1） |
| 4 | 22(3） | 36(1） | 24(1） | 42(1） | 21(1） |
| 5 | 35(2） | 30(1） | 20(1） | 37(1） | 21(1） |
| 6 | 18(4） | 33(2） | 23(1） | 37(1） | 22(1） |
| 7 | 28(3） | 32(1） | 26(1） | 35(1） | 21(1） |
| 8 | 19(2） | 36(1） | 18(1） | 42(1） | 30(1） |
| $\overline{\text{x}}\pm\text{s}$ | 26.3±8.1  (3.3±1.4) | 32.0±5.0  (1.1±0.4) | 25.1±5.8 (1.0±0.0) | 36.3±4.6  (1.0±0.0) | 24.3±3.8  (1.0±0.0) |

a(b): Sleeping time (Induction time)

*After the acupuncture response recovered, rabbits in low-dose and high-dose groups were intravenously injected with a mixed solution containing 0.15 mL/kg brimonidine and chloral hydrate, and then re-sleeping was observed.
