# Supplementary material for: Preliminary evaluation of the efficacy and safety of brimonidine for general anesthesia
Source: BMC Anesthesiol. 2021 Dec 3;21:305. doi: 10.1186/s12871-021-01516-1 (PMC8641169; doi:10.1186/s12871-021-01516-1)
Supplement: Supplementary file 10 — Additional file 10: Table 10. Brimonidine concentrations in the plasma after the intravenous administration (mg/ml). [file 12871_2021_1516_MOESM10_ESM.docx]

**Additional file 10**

Table10 Brimonidine concentrations in the plasma after the intravenous administration（mg/ml）

|  | 10min | 15min | 30min | 45min | 60min | 90min | 120min |
| --- | --- | --- | --- | --- | --- | --- | --- |
| 1 | 0.0637 | 0.0611 | 0.0434 | 0.0364 | 0.0135 | 0.0130 | 0.0105 |
| 2 | 0.0593 | 0.0791 | 0.0690 | 0.0350 | 0.0153 | 0.0141 | 0.0119 |
| 3 | 0.0906 | 0.0710 | 0.0695 | 0.0474 | 0.0126 | 0.0128 | 0.0135 |
| $\overline{\text{x}}\pm\text{s}$ | 0.0712±  0.0169 | 0.0704±  0.0090 | 0.0606±  0.0149 | 0.0396±  0.0068 | 0.0138±  0.0014 | 0.0133±0.0007 | 0.0120±  0.0015 |
